# A Readily Applicable Strategy to Convert Peptides to Peptoid-based Therapeutics

**DOI:** 10.1371/journal.pone.0058874

**Published:** 2013-03-21

**Authors:** Minyoung Park, Modi Wetzler, Theodore S. Jardetzky, Annelise E. Barron

**Affiliations:** 1 Department of Chemical and Systems Biology, School of Medicine, Stanford University, Stanford, California, United States of America; 2 Department of Bioengineering, Schools of Engineering and Medicine, Stanford University, Stanford, California, United States of America; 3 Department of Structural Biology, School of Medicine, Stanford University, Stanford, California, United States of America; University of Waterloo, Canada

## Abstract

Incorporation of unnatural amino acids and peptidomimetic residues into therapeutic peptides is highly efficacious and commonly employed, but generally requires laborious trial-and-error approaches. Previously, we demonstrated that C_20_ peptide has the potential to be a potential antiviral agent. Herein we report our attempt to improve the biological properties of this peptide by introducing peptidomimetics. Through combined alanine, proline, and sarcosine scans coupled with a competitive fluorescence polarization assay developed for identifying antiviral peptides, we enabled to pinpoint peptoid-tolerant peptide residues within C_20_ peptide. The synergistic benefits of combining these (and other) commonly employed methods could lead to a easily applicable strategy for designing and refining therapeutically-attractive peptidomimetics.

## Introduction

Synthetic peptides are highly attractive therapeutic candidates [1], [2]. However, challenges in bringing these peptides to broader clinical use still remain due to rapid renal clearance, relatively low cell permeability, variable solubility in physiological conditions, and limited proteolytic stability [3], [4]. Peptides containing peptidomimetic residues can reap the advantages of synthetic peptides while minimizing the problematic pharmacological properties of parent peptides [5]. Among the many peptidomimetic classes, peptoids are readily synthesized and sequence-specific bioinspired oligomers of *N*-substituted glycines [6]. Because peptoid side chains are attached to the backbone nitrogen instead of the α-carbon, peptoids are highly resistant to proteolysis, providing vast opportunity for biomedical aaplications [6]–[12].

The simplest way to convert bioactive peptides to peptoid analogues should be the substitution of amino acids in the peptide sequence by peptoid monomers with identical or similar side chains. However, because biological function can be easily influenced by even minor structural changes, often many variants have to be individually synthesized, tested, and progressively optimized, yielding limited success in transforming therapeutically interesting peptides into peptoids [13]–[17]. In contrast, a rigorous approach to identify positions for peptoid substitutions would accelerate efforts to incorporate peptoid residue into targeted peptides. Notably, an almost limitless variety of side chains in peptoid residues [18] can be introduced by the submonomer synthesis approach [19], which allows inexpensive but extensive optimization of peptoid residues [20], [21]. Consequently, developing an unbiased and broadly-applicable approach to guide the conversion of bioactive peptides into peptoids or peptide-peptoid conjugates (peptomers) is essential and could be a game-changing, enabling technology in creating better peptoid-based therapeutics.

**Figure 1:**
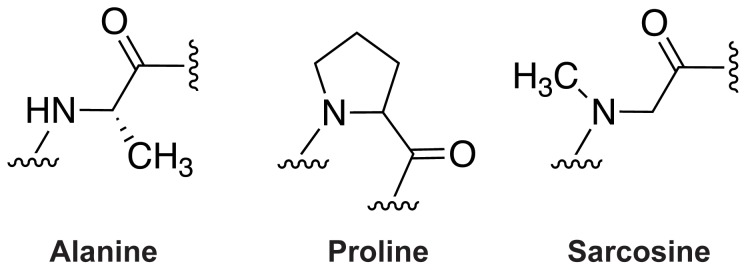
Structural comparison of alanine, proline and sarcosine.

When substituting individual residues in peptides with regular amino acids, an “alanine scan” is the most conventional method to determine the relative importance of each side chain in biological function [22]. In this procedure, the impact of alanine-substituted variants can be assessed. In replacing peptide residues with peptidomimetics, however, the peptide backbone itself is typically altered, and thus several prerequisites must be met. Specifically, we propose the following three criteria that must be assessed to achieve successful substitution of peptoid residues into peptides: first, the importance of the original amino acid side chain, as can be elucidated with an **alanine scan**; second, the importance of the amide hydrogen, which is missing in peptoids, and could be investigated using **sarcosine** (Figure 1), as the peptoid equivalent of alanine; third, particularly if substituting peptoid residues into a helix, the localized tolerance of the peptide α–helix for the peptoid polyproline type I (PPI)-like helix structure needs to be assessed. Since peptoid helices form a PPI-like helix [23], **proline** itself could quite reasonably serve as a probe of the suitability of the parent helix to incorporate this structure. Whereas the alanine scan has been routinely applied, reports of either proline [24], [25] or sarcosine scans are rare. Additionally there are no reports of combining these approaches.

In this study, we employ a readily applicable approach for fast identification of peptoid-tolerant residues in a target peptide sequence by combining alanine, proline and sarcosine scans. In previous studies of human Respiratory Syncytial Virus (hRSV) entry inhibition, we identified a peptide lead that is composed of 20 amino acids (C_20_) and evaluated its inhibitory activity by a protein-based competitive fluorescence polarization (FP) assay [26]. Using the C_20_ peptide as a parent template, each amino acid was successively substituted by alanine, proline and sarcosine and the binding affinity of these peptides to the target protein was individually examined. We identified several potentially peptoid-replaceable residues and replaced these pre-determined peptide residues with peptoid residues. To further prove the potential benefits of this approach, we introduced structural constraints into the resulting peptomers by either peptide side chain hydrocarbon stapling or peptoid side chain click chemistry to stabilize the helical conformation.

Here we revisit the alanine, proline, and sarcosine scans and reevaluate their potential as a comprehensive strategy for designing therapeutic peptoids and peptoid-peptide hybrids (peptomers). Furthermore, our approach could be applied to other peptidomimetic systems with altered peptide backbones, such as by combining alanine and β–alanine scans to generate β–peptide containing therapeutic peptides, or by combining multiple such assays (e.g., alanine, sarcosine, and β–alanine scans) to determine the best peptidomimetic substitutions for any position in therapeutic peptides.

## Materials and Methods

### General materials

Chemical reagents for peptide and peptomer synthesis were purchased from Applied Biosystems (Foster city, CA, USA) or Sigma-Aldrich (Milwaukee, WI, USA) and used without further purification. Resins and Fmoc*-*protected amino acids were purchased from Novabiochem (San Diego, CA, USA), AAPPTEC (Louisville, KY, USA), or Anaspec (San Jose, CA, USA). Solvents and reagents for peptoid monomer synthesis and for analytical and preparative RP-HPLC were purchased from Fisher Scientific (Pittsburgh, PA, USA). All other chemicals were purchased from Sigma-Aldrich (Milwaukee, WI, USA) and used without additional purification.

### Alanine, proline, and sarcosine-substituted peptides preparation

Peptides with alanine, proline, and sarcosine substitution were commercially obtained with 95% purity (AAPPTEC, Louisville, KY, USA) and their sequences are listed in Table S1. Final purities and the molecular weight of the synthetic peptides were confirmed by analytical RP-HPLC and electrospray ionization mass spectrometry (ESI).

### Synthesis of peptomeric C_20_ analogues

Peptomeric C_20_ analogues were synthesized in the laboratory using standard Fmoc chemistry for peptide residues and submonomer peptoid synthesis[19] for peptoid residues using an automated ABI 433A peptide synthesizer (Applied Biosystems, Foster city, CA, USA). After synthesis, the peptides were cleaved from the resin and deprotected in trifluoroacetic acid (TFA)/water/TIPS/thioanisole (90∶5∶2.5∶2.5 v/v) for 1.5 hr at room temperature. Peptomers were purified by preparative RP-HPLC on a C18 column using a linear gradient of 5–99% solvent B in solvent A over 60 min (solvent A is 0.1% (v/v) TFA in water and solvent B is 0.1% (v/v) TFA in acetonitrile). Final purities of the synthetic peptides were confirmed to be > 95% by analytical RP-HPLC, and the molecular weight of the purified product was confirmed by ESI at the Stanford University Mass Spectrometry (SUMS) facility.

### Microwave-assisted on-resin click chemistry

Preparation and purification of the peptomeric C_20_ analogues with clickable peptoid monomers (Figure S2) were performed in the laboratory. Click chemistry (1,3-dipolar cycloaddition) between the azide and alkyne functional groups was performed on resin. Stock solutions were prepared: (i) 1 M of CuSO_4_ 5H_2_O (Santa Cruz Biotechnology, Santa Cruz, CA, USA) was dissolved in H_2_O; (ii) 1M of *L*-(+)-sodium ascorbate (Santa Cruz Biotechnology, Santa Cruz, CA, USA) was dissolved in *t*-BuOH/H_2_O (1∶1). For a microwave-assisted reaction, 0.13 mmole of partially synthesized peptide-bound resin in 75 mL of t-BuOH/H_2_O (1∶1) was placed in the reaction tube and subsequently L-(+)-sodium ascorbate (520 µmol) and CuSO_4_ 5H_2_O (130 µmol) were added. The reaction tube was sealed and heated in the microwave reactor for 1 hr (70°C, absorption level: high). The reaction mixture was pale yellow after microwave heating. The clicked and unclicked peptomers were purified by preparation RP-HPLC on a C18 column using a linear gradient of 5–99% solvent B in solvent A over 60 min (solvent A is 0.1% (v/v) TFA in water and solvent B is 0.1% (v/v) TFA in acetonitrile) and their analytical HPLC traces are shown in Figure S1. To avoid undesirable truncated products, microwave-assisted on-resin click chemistry was carried out on partially synthesized peptomers first, and the rest of the synthesis was then completed. Microwave-assisted on-resin click chemistry was carried out after the 6^th^ residue to achieve high efficiency of the click reaction (Figure S3). The synthesis of the rest of the C_20_ peptide sequence was completed by using an automated ABI 433A peptide synthesizer. The completion of the click reaction was verified using a simple colorimetric method based on Kaiser test (Figure S4) [27]. For the unclicked versions, the syntheses were completed without interruption.

### Competitive fluorescence polarization (FP) binding assay

Competitive FP binding assays of the C_20_ derivatives were carried out as previously described [26]. Briefly, a 5-helix bundle (5HB) protein was designed to mimic a post-fusion structure of fusion protein F (6-helix bundle) and provide a specific binding site for screening potential human respiratory syncytial virus (hRSV) entry inhibitors. Using a 5HB protein as a target protein and a fluorescently labeled 35 amino acid-long peptide (Fl-C_35_, sequence shown in Table S1) as a tracer, we performed a competitive FP assay to evaluated the binding affinity of each C_20_ analogue prepared in this study. In each well of a black 96-well plate (Corning Inc. Lowell, MA, USA), 20 nM of the 5HB with increasing concentrations of each peptomer in FP buffer (20 mM PBS at pH 7.4, 500 mM NaCl, 0.01% (v/v) Tween*-*20, and 0.05 mg/ml bovine gamma globulin) in a final volume of 185 µL was incubated for 1h at room temperature. Fl-C_35_ was then added to a final concentration of 5 nM followed by 30 min incubation at room temperature. The FP responses were monitored using a Synergy4 plate reader (Biotek, Winooski, VT, USA) with an excitation wavelength of 485 nm and an emission wavelength of 530 nm. The percentage of inhibition (% Inhibition) was calculated from the equation: % inhibition  =  100×[1-(mP-mPf)/(mPb-mPf)]. Unbound Fl-C_35_ (mPf), bound Fl-C_35_ (mPb) and the bound inhibitor to the 5HB (mP) are used accordingly.

### Stapled peptides preparation

As a proof-of-concept experiment, a hydrocarbon-stapled peptide was designed. The stapling positions to incorporate unnatural amino acids that have both an α-methyl group and an α-alkenyl group (e.g., (*S*)-2-(4′-pentenyl) alanine) were determined based on the alanine, proline, and sarcosine scanning studies presented in the manuscript. A resulting peptide stapled at (*i*, *i* + 4) positions (ISQVNEKINQSLXFIRXSDE, X for hydrocarbon staples), SC_20_, was commercially obtained from Anaspec (San Jose, CA, USA) with final purity (> 90%).

### Secondary structural analysis by Circular Dichroism (CD) spectroscopy

CD spectra were obtained using a Jasco J-815 spectrophotometer (JASCO, Easton, MD, USA). Samples were prepared in water with 25% (v/v) acetonitrile with concentrations ranging from 25–50 µM. Data was collected from 195 to 260 nm with a step size of 0.2 nm, at a rate of 100 nm/min, a bandwidth of 1.0 nm with a scanning speed of 20 nm/min in a 0.1 cm path-length quartz cell at room temperature. The response time was 0.5 sec. Each CD spectrum was an average of 3 measurements and the baseline was corrected by subtracting the spectrum of a blank obtained under identical conditions. The resulting data was converted to per-residue molar ellipticity units (deg·cm^2^·dmol^−1^·residuel^−1^). The ratio of molar ellipticities at 208 and 222 nm was used to calculate the helical content of each C_20_ analogue. The secondary structure content was analyzed with a web-based software, K2D2 (http://www.ogic.ca/projects/k2d2).

## Results and Discussion

### Effects of Alanine Scanning

We previously developed a protein-based fluorescence polarization (FP) competitive assay for identifying specific antivirals that block viral fusion and subsequent entry using human respiratory syncytial virus (hRSV) fusion F protein as a representative of class I fusion protein-mediated viral entry [26]. Using this system, we demonstrated that a 20 amino acid-long peptide derived from the 6^th^ helix of hRSV fusion protein F (C_20_) retains weakened but moderate binding affinity to a target protein, Since the C_20_ peptide is the shortest hRSV F-derived peptide with measurable biological activity, we sought to further examine the determinants of biological activity of the C_20_ peptide at a residue level and to seek a way of improving its target protein-binding affinities by applying our own expertise on designing peptidomimetics using peptoids.

**Figure 2:**
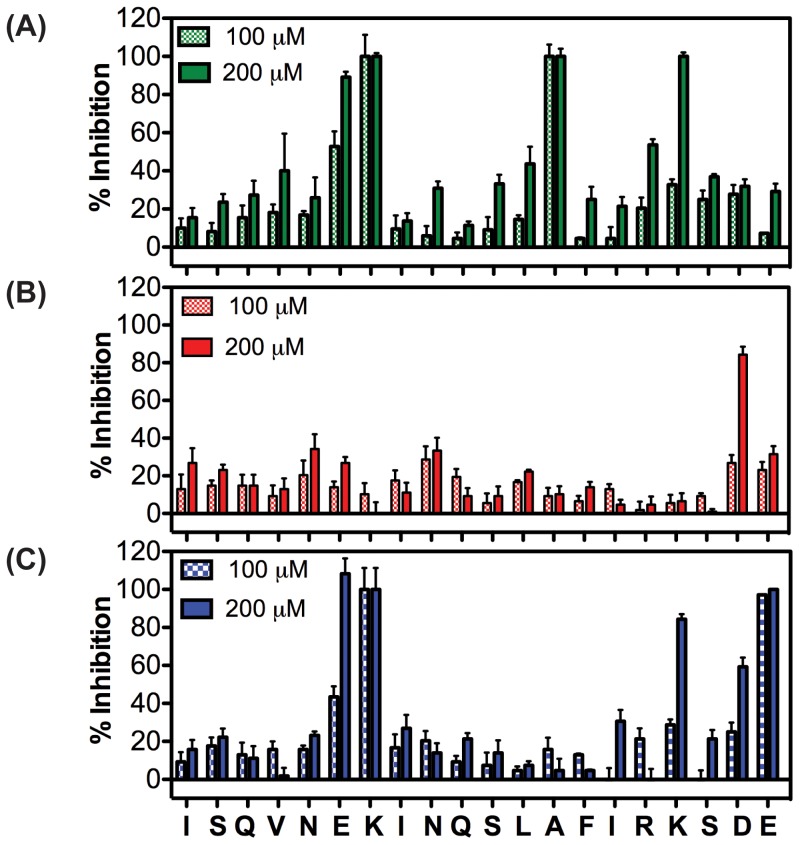
% Inhibition of (A) alanine-, (B) proline-, and (C) sarcosine-substituted peptides tested at 100 and 200 µM using a 5HB-based competitive FP assay.

Twenty singly alanine-substituted C_20_ peptides (Table S1) were examined to understand the importance of the side chains in the interaction of the C_20_ peptide with a target protein (5HB). The binding affinity of each peptide to the 5HB was measured at concentrations of 100 and 200 µM using a protein-based competitive FP assay as previously reported; tighter binding indicates that replacement of the amino acid side chain with alanine did not greatly impact the binding affinity [26]. The results, shown as % inhibition of the Fl-C_35_ tracer binding to the 5HB, are presented in Figure 2A. Alanine substitutions at Glu^6^, Lys^7^, and Lys^17^ appear to have minimal impact on the binding affinity to the target. This is not surprising because according to the crystal structure of the hRSV F protein [28], these amino acid residues are located away from the expected binding interface, implying that these peptide residues could be candidates for peptoid substitution. Our observations are consistent with the extreme thermal and structural stability of the helical bundle to denaturing condiditons [26] and suggest that the hydrophobic residues involved in the direct interaction between the C_20_ peptide and the 5HB are critical for binding of the C_20_ peptide to the 5HB and should not be altered.

### Effects of Proline Scanning

The only naturally occurring *N*-substituted amino acid, proline, has been recognized as a natural candidate for peptoid substitutions in various biological systems. For example, prolines in WW and SH3 domain-binding peptides have been replaced with peptoid residues resulting in improved binding affinity [29]. In addition, proline substitutions with peptoid monomers have enhanced the antimicrobial activity of proline-containing antimicrobial peptides [30], [31] and binding affinity of plant peptide hormones [12]. Therefore, we decided to substitute each amino acid in the C_20_ peptide with proline (Table S1) and assumed that this proline substitution would be a straightforward way of directly identifying peptoid-tolerant peptide residues. Not unexpectedly, proline-substitution deleteriously affected the binding affinities of the C_20_ peptide analogues, yielding low % inhibition values even at 200 µM (Figure 2B).

Although substituting proline residues with peptoids is a proven strategy, it is noteworthy that the use of prolines as representatives of peptoid substitution is more complex since their highly constrained structures only represent a subset of possible peptoid conformations and side chains, such as those that promote the PPI-like structure. As a potential advantage, such sterically constrained peptoid side chains could be entropically favorable leading to improved binding affinity. Alternatively, the occasional unusual conformations adopted by proline [32], its significant flexibility which generates a kink along the α-helix [33]–[35], or rapid *cis*/*trans* isomerization [36] can have negative consequences for binding affinity. It is postulated that perturbation of structural features of the α-helix in these proline-substituted C_20_ peptide predominates, thereby interfering in binding of the proline-substituted C_20_ peptides to the target. The difficulty of substituting a peptoid residue into an α-helix has been also recently reported [17].

The findings from the proline substitution studies on the C_20_ peptide confirmed that maintaining the α-helical conformation is crucial for the C_20_ peptide to bind to the 5HB and thus peptomers with α-chiral peptoid residues were not synthesized. Notably, this is the first time that the hypothesis of using proline as a representative of structure-promoting peptoids has been systematically tested in the context of designing peptoid-based peptidomimetics from peptides that do not naturally contain prolines. In addition, the greater rigidity of the α-chiral peptoid residues could be beneficial to improving the binding affinity of non-α -helical peptides, indicating that a proline scan would be more relevant in such systems.

### Effects of Sarcosine Scanning

Sarcosine, also known as *N*-methylglycine, is the simplest peptoid monomer, but lacks the structural restrictions on backbone angles of the cyclic proline residue. A sarcosine scan has been used to identify pharmacophores in peptoid ligands as a direct parallel to the alanine scan in peptides [37]. In contrast, we utilized the sarcosine substitution approach to reveal which peptide residues can be replaced with peptoid monomers. Twenty sarcosine-substituted peptides using the C_20_ peptide as a template were commercially obtained (Table S1) and their binding affinities to the 5HB were measured in the 5HB-based competitive FP assays (Figure 2C). These results reveal that sarcosine substitutions of Glu^6^, Lys^7^, Lys^17^, Asp^19^, and Glu^20^ minimally disrupted the original binding activity of the parental peptide. The resulting data indicate the importance of the amide hydrogens in the backbone of the C_20_ peptide to maintain its binding affinity to the 5HB directly. While proline has previously been used to indicate sites of potential substitution by structure-promoting peptoid residues, this data suggest the sarcosine scan as a more effective screen for evaluating the substitution of peptide residues by peptoid monomers.

### Synthesis and characterization of peptomeric C_20_ analogues

**Figure 3:**
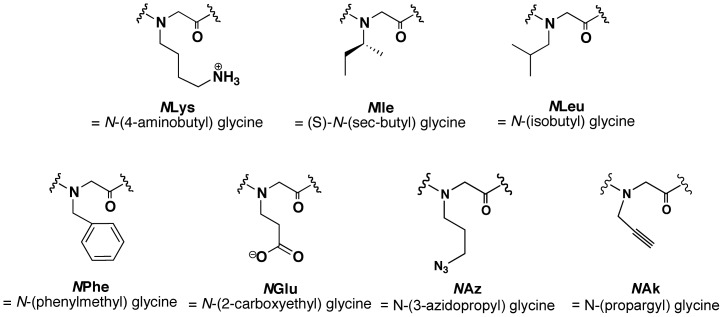
Structures of peptoid residues corresponding to structurally identical peptides used for C_20_ peptomeric analogues synthesis and clickable peptoid residues used for clicked C_20_ analogues.

**Figure 4:**
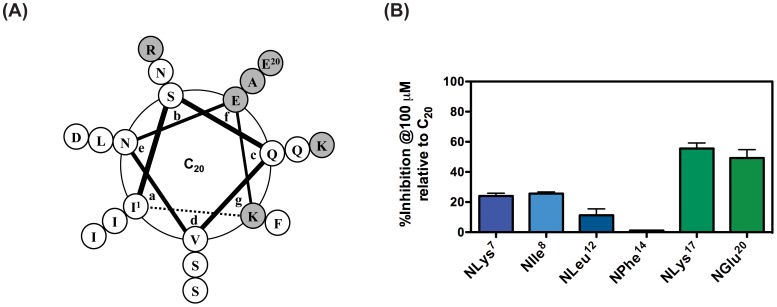
Helical wheel of C_20_ peptide and relative % inhibition of singly substituted peptomers. (A) Peptide residues identified as tolerating positions for peptoid substitution are shown in shaded circle. The heptad position is based on hRSV F fusion protein sequence. (B) Based on the results shown in panel (A), peptomers with single peptoid substitution were prepared and their relative % inhibition to C_20_ peptide at 100 µM were examined using a 5HB-based competitive FP assay.

As a proof of concept to experimentally confirm the utility of the alanine, proline, and sarcosine scan results in designing peptidomimetics, we replaced chosen peptide residues in the C_20_ peptide with peptoid monomers containing structurally identical side chains (Figure 3) to design peptomers (i.e., peptoid-peptide hybrids). Three residues (Lys^7^, Lys^17^, and Glu^20^) were selected as representatives of peptoid-tolerant positions and three other non-tolerant peptide residues (Ile^8^, Leu^12^, and Phe^14^) were chosen to serve as negative controls (Figure 4A, Table 1). The binding affinity of each peptomeric C_20_ analogue to the 5HB was measured using the 5HB-based competitive FP assay. The resulting % inhibition values calculated from polarization values of individual peptomer at 100 µM are presented in Figure 4B, proving the potential of the combined scanning approach as a tool for converting therapeutic peptides to peptoid-substituted peptidomimetics.

**Table 1:** Sequences of C_20_ peptide and its peptomeric analogues.

Oligomers	Sequences (amino to carboxyl)
C_20_	ISQVNEKINQSLAFIRKSDE
*N*Lys^7^	ISQVNE*N*LysINQSLAFIRKSDE
*N*Ile^8^	ISQVNEK*N*IleNQSLAFIRKSDE
*N*Leu^12^	ISQVNEKINQS*N*LeuAFIRKSDE
*N*Phe^14^	ISQVNEKINQSLA*N*PheIRKSDE
*N*Lys^17^	ISQVNEKINQSLAFIR*N*LysSDE
*N*Glu^20^	ISQVNEKINQSLAFIRKSD*N*Glu

The peptoid-tolerant residues identified from this study are shown in bold and underlined.

More specifically, peptomers with peptoid-tolerant positions led to an about 3-fold better inhibition in selected cases (average % inhibition  =  52.4% for *N*Lys17 and *N*Glu20) relative to peptoid substitutions at control non-peptoid-tolerant residues (average % binding  =  18.4 % for *N*Ile8 and *N*Leu12). Peptide residues closely involved in hydrophobic interactions with neighboring helices (i.e., *a* and *d* position) are clearly not favorable for peptoid substitution in any cases. Surprisingly, this relationship also holds true for salt bridge interactions. For example, *N*Lys peptoid substitution at Lys^7^, which is likely involved in an interhelical salt bridge, seems to be more deleterious than *N*Lys substitution at Lys^17^ in terms of the binding affinity to the 5HB. The binding affinity of the peptomer with *N*Phe at Phe^14^ could not be measured due to its poor solubility, but we observed different responses for peptoid substitutions at Ile8 and Leu^12^, even though both positions were predicted as non-tolerating residues for peptoid replacement. We believe that peptide residues located within the helical core of an &alpha;-helix should be kept intact and are extremely sensitive to peptoid substitution (e.g., Leu^12^), because the system used in this study is based on the interaction between α-helices. Our results demonstrate the combined scanning approach can improve biological functions for rationally designed peptomers, solely designed by the systematic scans relative to randomly substituted peptomers.

### Structurally constrained C_20_ peptide derivatives via click chemistry reaction

Short peptides usually show ill-defined 3D-structures in aqueous solutions, implying that there are an immense number of conformational isomers present whose energy loss upon binding is entropically unfavorable. Likewise, the C_20_ peptide is known to be poorly structured by itself [38], [39] and consequently this unstructured conformation of C_20_ peptide may lead to high susceptibility to proteolytic degradation. We thus further explored the potential of intramolecular cyclic C_20_ peptide analogues via copper-catalyzed azide-alkyne cycloadditions (i.e. click chemistry) to stabilize the helical structures [40]–[43]. We therefore synthesized structurally constrained peptomeric C_20_ analogues between clickable peptoid residues (*N*Az and *N*Ak illustrated in Figure 3) using on-resin click reaction (Table 2). Since selected residues are at (*i*, *i* + 4) positions, it is considered that at least one-turn of the helix can be formed. It is well known internal and terminal structural constraints in peptides can lead to markedly different effects [44]. We therefore wanted both an internal and an external structural constraint. Residue Ala^13^ (*i* position) although not identified in the sarcosine scan as peptoid tolerant, was selected as the most likely to provide a reasonable internal structural constraint in combination with the highly peptoid-tolerant Lys^17^ (*i* + 4 position). The resulting peptomers tested in this study are following: terminally clicked C1C_20_, internally clicked C2C_20_ along, and their unclicked counterparts (UC1C_20_ and UC2C_20_) as controls (Table 2 and Figure S2).

**Table 2:** Peptomers tested in this study.

Oligomers	Sequences (amino to carboxyl)
*N*Lys^7^ *N*Glu^20^	ISQVNE*N*LysINQSLAFIRKSD*N*Glu
C1C_20_	ISQVNEKINQSLAFI*N*AzKSD*N*Ak
C2C_20_	ISQVNEKINQSL*N*AzFIR*N*AkSDE

Terminally and internally clicked peptomers between *N*Az and *N*Ak are shown along with doubly substituted peptomer as a negative control.

**Figure 5:**
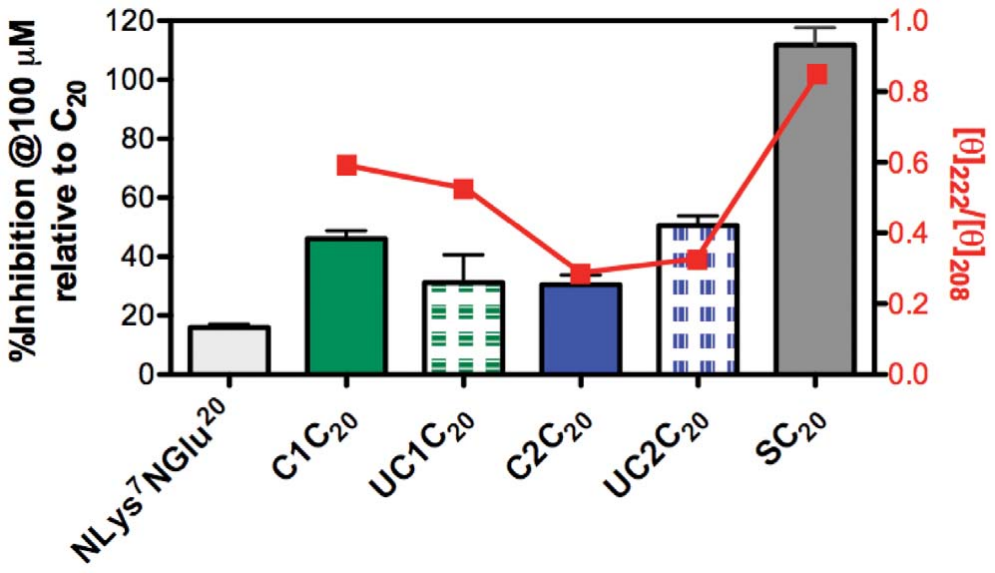
Relative % inhibition and ratio of molar ellipticities at 222 and 208 nm. Terminally and internally clicked (C1C_20_, C2C_20_) and unclicked (UC1C_20_, UC2C_20_) peptomers and a stapled peptide analog (SC_20_) as a positive control were tested. % Inhibition of structurally constrained C_20_ analogues relative to C_20_ peptide were measured at 100 µM shown in bars. The ratio of molar ellipticities (unit: deg·cm^2^·dmole^-1^) at 222 and 208 nm is used to evaluate the helical content in each peptomer and is shown in red line (left y-axis).

We evaluated potential improvements in binding of the clicked peptomers to the target protein compared with the unclicked peptomers and the parent peptide (C_20_) using the 5HB-based competitive FP assays described previously (Figure 5). We postulated that the clicked peptomers would more readily adopt conformations thereby showing tighter binding affinity to the 5HB, although structural constrains can sometimes lead to lower binding affinity due to populating a highly structured but inactive conformation [44]. Both clicked peptomers exhibited better binding than a doubly peptoid substituted control peptomer, *N*Lys^7^ *N*Glu^20^. Surprisingly, however, both the terminally and internally clicked peptomers did not exceed the binding ability of C_20_ to the 5HB, thus suggesting the constrained peptomers were not constrained into an ideal binding geometry. This hypothesis is consistent with the observation that the unclicked UC2C_20_ is better than the clicked C2C_20_. Even so, the terminally constrained peptomer showed the tightest binding to the 5HB amongst all the peptomers tested in our study. Together, these results suggest that while the peptoid substitution disrupts the hydrogen bonding necessary for α-helix formation, the structural constraint introduced by clicking allow us to moderately recover the binding activity relative to a doubly substituted control peptomer.

## Conclusions

Several attempts to transform therapeutic peptides to biologically active peptoid-based peptidomimetics have yielded encouraging but rather limited success. We thus propose a readily-applicable strategy for identifying peptoid-replaceable peptide residues, which explores the conformational tolerance and importance of individual side chains in the target peptide sequence, providing information that is not provided by structural studies (e.g., X-ray crystallography and NMR). The results we obtained through the combined scanning approach, presented in Figure 4A, suggest that peptide residues facing away from the binding site are likely candidates for peptoid replacement and further structural modifications. However, it is noteworthy that not all peptide residues located away from the binding groove are peptoid-tolerant, thus proving the usefulness of our combined scanning approach in determining optimal positions for peptoid incorporation. Based on the resulting data, singly and doubly-substituted peptomers were synthesized and evaluated, leading to moderate loss of binding affinity that could potentially be compensated for by increased protease resistance and prolonged life-time.

Furthermore, we explored two different strategies to stabilize the α-helical conformation of the C_20_ peptide via either hydrocarbon stapling between non-natural amino acids with an olefinic side chain or click chemistry cyclization between clickable peptoid residues. The clicked C_20_ analogues, C1C_20_ and C2C_20_, were also more α-helical than their unclicked versions and have tighter target binding relative to the control, a doubly substituted peptomer, *N*Lys^7^ *N*Glu^20^ (Figure 5, right y-axis). These results are consistent with our observations in the 5HB-based FP assays. This system, which predominantly consists of an α-helix target ligand, is perhaps suboptimal in evaluating the full value of the combined scanning approach to develop peptoid-based peptidomimetics. Protein interactions through α-helices require a precise spacing between peptide residues from each binding partner, with less than ideal tolerance of incorporating peptoids into the peptide sequences (e.g., the conformational difference of the peptoid helix and α-helix). Beyond the immediate application to the hRSV system reported here, we anticipate that the strategy presented here will be generally more informative and powerful in other peptide ligand systems (e.g., β-turn mimics and miniature protein scaffolds), providing a critical design paradigm for future therapeutic peptidomimetics.

Although one could have theoretically utilized the peptoid residues directly with the same side chains instead of sarcosine in a “peptoid scan”, we intentionally used the sarcosine scan for two reasons. First, although peptoid synthesis is entirely compatible with peptide synthesis, it is not as universally used; in contrast, sarcosine is commercially available as Fmoc-sarcosine and could thus be readily used in solid phase peptide synthesis protocols, thereby enabling wider adoption of this scanning technique. Second, although not pursued in this study, the data from this combined approach could also serve as a starting point for a library screening approach that would investigate peptoid side chains different from those of the original amino acid being substituted. Such an approach would take advantage of the near limitless chemical diversity of peptoids, which have already been synthesized with hundreds of different side chains [18]. The extension of this systematic scanning approach to other peptidomimetic systems with altered peptide backbones could also prove useful, such as by combining alanine, sarcosine and β–alanine scans of promising therapeutic peptides to determine the best class of peptidomimetic substitutions for any position in the therapeutic peptide.

## Supporting Information

Figure S1 **Analytical RP-HPLC traces for (A) terminally and (B) internally clicked and unclicked peptomers.**
(TIF)

Figure S2 **Structures of (A) terminally and (B) internally clicked (C1C_20_, C2C_20_) and unclicked (UC1C_20_, UC2C_20_) peptomers with desired molecular weights.** Azide and alkyne-bearing residues are presented in red and blue, respectively.
(TIF)

Figure S3 **Microwave-assisted Cu (II) catalyzed click chemistry on resin.** Azido and alkyne functional groups in peptoid side chains are shown in red and blue, respectively. To enhance the reaction efficiency of the click chemistry, on-resin click chemistry using microwave is adopted in this study.
(TIF)

Figure S4 **Modified Kaiser method.** To determine the completion of the click reaction on resin without additionally purification procedure, the modified Kaiser method was used. The synthesis of the terminally clicked peptomeric analogue was used as an example. (TPP; triphenyphospine).
(TIF)

Table S1 **Sequences of alanine-, proline-, and sarcosine-substituted peptides tested in this study along with a parent peptide, C_20_ and a tracer Fl-C_35_.** Their inhibitory effects on binding of Fl-C_35_ to the 5HB are presented as % inhibition at 200 µM.
(DOC)

Methods S1 **Additional experimental methods.**
(DOC)
