# Supplementary figures and images for: A Readily Applicable Strategy to Convert Peptides to Peptoid-based Therapeutics
Source: PLoS One. 2013 Mar 21;8(3):e58874. doi: 10.1371/journal.pone.0058874 (PMC3605428; doi:10.1371/journal.pone.0058874)

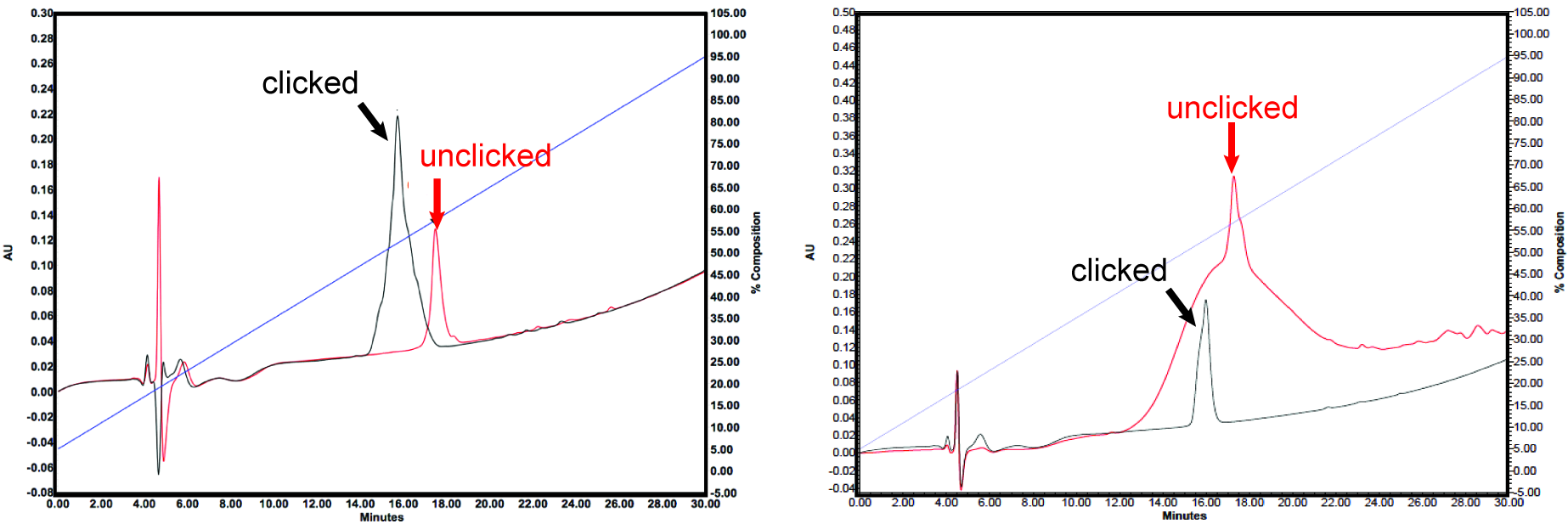

Supplement: Figure S1 — Analytical RP-HPLC traces for (A) terminally and (B) internally clicked and unclicked peptomers. (TIF) [file pone.0058874.s001.tif]

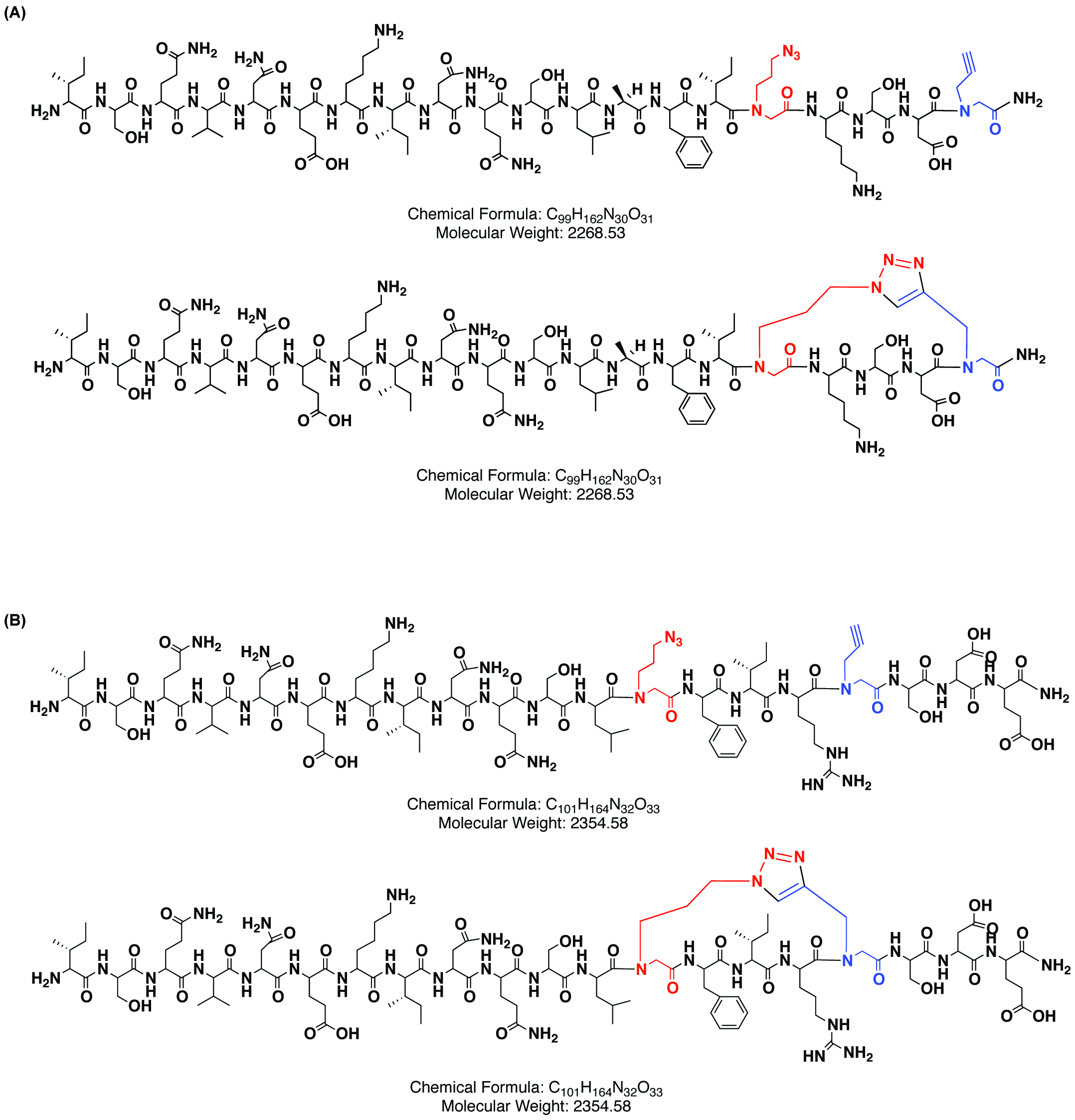

Supplement: Figure S2 — Structures of (A) terminally and (B) internally clicked (C1C20, C2C20) and unclicked (UC1C20, UC2C20) peptomers with desired molecular weights. Azide and alkyne-bearing residues are presented in red and blue, respectively. (TIF) [file pone.0058874.s002.tif]

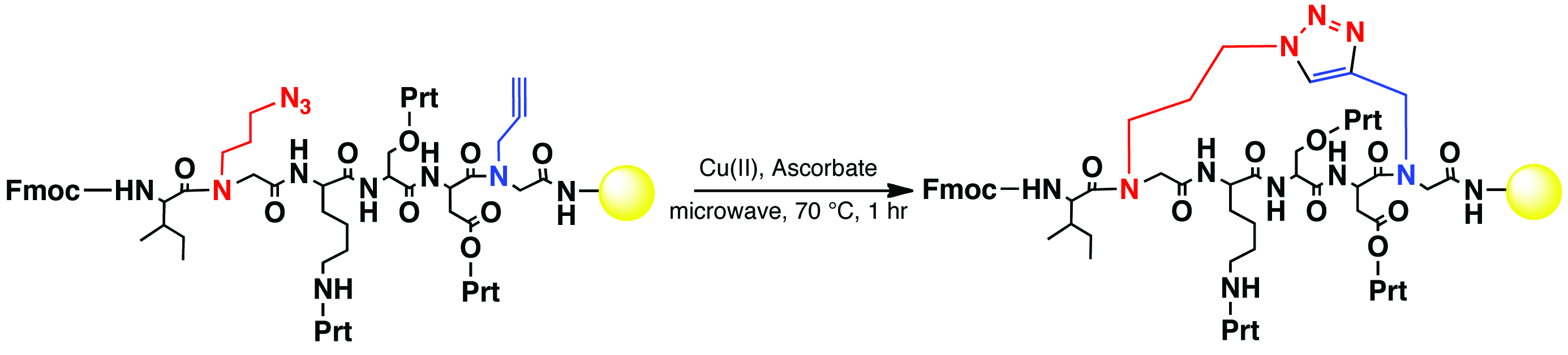

Supplement: Figure S3 — Microwave-assisted Cu (II) catalyzed click chemistry on resin. Azido and alkyne functional groups in peptoid side chains are shown in red and blue, respectively. To enhance the reaction efficiency of the click chemistry, on-resin click chemistry using microwave is adopted in this study. (TIF) [file pone.0058874.s003.tif]

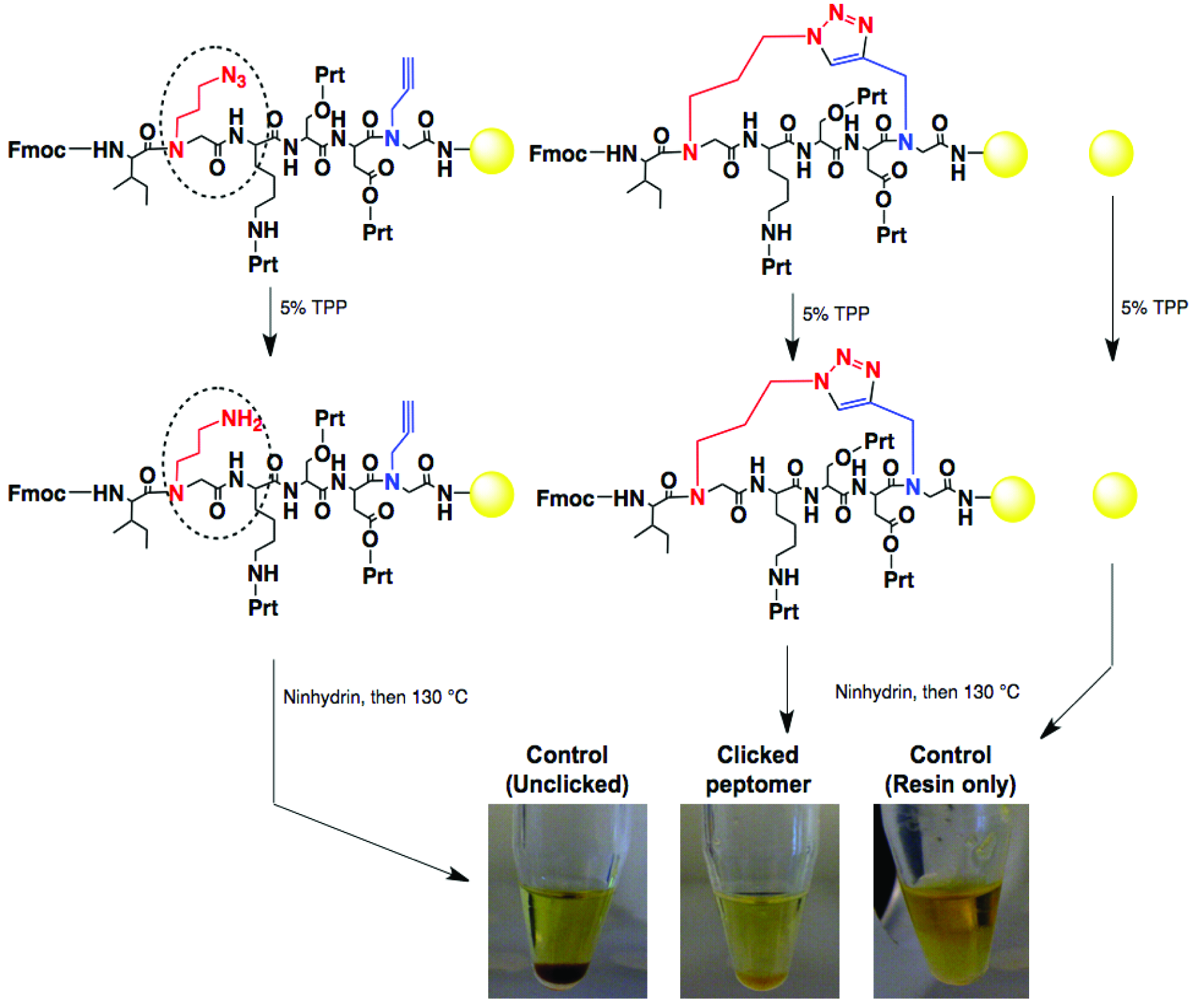

Supplement: Figure S4 — Modified Kaiser method. To determine the completion of the click reaction on resin without additionally purification procedure, the modified Kaiser method was used. The synthesis of the terminally clicked peptomeric analogue was used as an example. (TPP; triphenyphospine). (TIF) [file pone.0058874.s004.tif]
