# Supplementary material for: A Readily Applicable Strategy to Convert Peptides to Peptoid-based Therapeutics
Source: PLoS One. 2013 Mar 21;8(3):e58874. doi: 10.1371/journal.pone.0058874 (PMC3605428; doi:10.1371/journal.pone.0058874)
Supplement: Table S1 — Sequences of alanine-, proline-, and sarcosine-substituted peptides tested in this study along with a parent peptide, C20 and a tracer Fl-C35. Their inhibitory effects on binding of Fl-C35 to the 5HB are presented as % inhibition at 200 µM. (DOC) [file pone.0058874.s005.doc]

| **Peptide** | **Sequences** | **MW (Da)** | **% Inhibition at 200 μM** |
| --- | --- | --- | --- |
| **Fl-C_35_** | FITC-YDPLVFPSDEFDASISQVNEKINQSLAFIRKSDEL | **4016.4** | **100** |
| **C_20_** | **ISQVNEKINQSLAFIRKSDE** | **2319.6** | **100** |
| A1 | **A**SQVNEKINQSLAFIRKSDE | 2277.5 | 15.45 |
| A2 | I**A**QVNEKINQSLAFIRKSDE | 2304.6 | 23.63 |
| A3 | IS**A**VNEKINQSLAFIRKSDE | 2263.5 | 27.27 |
| A4 | ISQ**A**NEKINQSLAFIRKSDE | 2291.8 | 40.00 |
| A5 | ISQV**A**EKINQSLAFIRKSDE | 2277.6 | 25.90 |
| A6 | ISQVN**A**KINQSLAFIRKSDE | 2261.6 | 89.09 |
| A7 | ISQVNE**A**INQSLAFIRKSDE | 2262.5 | 100.00 |
| A8 | ISQVNEK**A**NQSLAFIRKSDE | 2277.5 | 13.63 |
| A9 | ISQVNEKI**A**QSLAFIRKSDE | 2276.6 | 30.90 |
| A10 | ISQVNEKIN**A**SLAFIRKSDE | 2262.6 | 11.36 |
| A11 | ISQVNEKINQ**A**LAFIRKSDE | 2303.6 | 33.18 |
| A12 | ISQVNEKINQS**A**AFIRKSDE | 2277.5 | 43.63 |
| A13 | ISQVNEKINQSL**A**FIRKSDE | 2319.6 | 100.00 |
| A14 | ISQVNEKINQSLA**A**IRKSDE | 2243.5 | 25.00 |
| A15 | ISQVNEKINQSLAF**A**RKSDE | 2277.5 | 21.36 |
| A16 | ISQVNEKINQSLAFI**A**KSDE | 2234.5 | 53.63 |
| A17 | ISQVNEKINQSLAFIR**A**SDE | 2262.5 | 100.00 |
| A18 | ISQVNEKINQSLAFIRK**A**DE | 2303.6 | 36.81 |
| A19 | ISQVNEKINQSLAFIRKS**A**E | 2275.6 | 31.81 |
| A20 | ISQVNEKINQSLAFIRKSD**A** | 2261.6 | 29.09 |
| P1 | **P**SQVNEKINQSLAFIRKSDE | 2303.5 | 26.85 |
| P2 | I**P**QVNEKINQSLAFIRKSDE | 2329.6 | 23.15 |
| P3 | IS**P**VNEKINQSLAFIRKSDE | 2288.5 | 14.81 |
| P4 | ISQ**P**NEKINQSLAFIRKSDE | 2317.5 | 12.96 |
| P5 | ISQV**P**EKINQSLAFIRKSDE | 2302.6 | 34.26 |
| P6 | ISQVN**P**KINQSLAFIRKSDE | 2287.6 | 26.85 |
| P7 | ISQVNE**P**INQSLAFIRKSDE | 2288.5 | - |
| P8 | ISQVNEK**P**NQSLAFIRKSDE | 2303.5 | 11.11 |
| P9 | ISQVNEKI**P**QSLAFIRKSDE | 2302.6 | 33.33 |
| P10 | ISQVNEKIN**P**SLAFIRKSDE | 2288.5 | 9.26 |
| P11 | ISQVNEKINQ**P**LAFIRKSDE | 2329.6 | 9.26 |
| P12 | ISQVNEKINQS**P**AFIRKSDE | 2303.5 | 22.22 |
| P13 | ISQVNEKINQSL**P**FIRKSDE | 2345.6 | 10.19 |
| P14 | ISQVNEKINQSLA**P**IRKSDE | 2269.5 | 13.89 |
| P15 | ISQVNEKINQSLAF**P**RKSDE | 2303.5 | 4.63 |
| P16 | ISQVNEKINQSLAFI**P**KSDE | 2260.5 | 4.63 |
| P17 | ISQVNEKINQSLAFIR**P**SDE | 2288.5 | 6.48 |
| P18 | ISQVNEKINQSLAFIRK**P**DE | 2329.6 | 0.93 |
| P19 | ISQVNEKINQSLAFIRKS**P**E | 2301.6 | 84.26 |
| P20 | ISQVNEKINQSLAFIRKSD**P** | 2287.6 | 31.48 |
| Sar1 | **Sar**SQVNEKINQSLAFIRKSDE | 2277.5 | 15.74 |
| Sar2 | I**Sar**QVNEKINQSLAFIRKSDE | 2303.6 | 22.22 |
| Sar3 | IS**Sar**VNEKINQSLAFIRKSDE | 2262.6 | 11.11 |
| Sar4 | ISQ**Sar**NEKINQSLAFIRKSDE | 2291.6 | 1.85 |
| Sar5 | ISQV**Sar**EKINQSLAFIRKSDE | 2276.6 | 23.15 |
| Sar6 | ISQVN**Sar**KINQSLAFIRKSDE | 2261.6 | 108.33 |
| Sar7 | ISQVNE**Sar**INQSLAFIRKSDE | 2262.6 | 100.00 |
| Sar8 | ISQVNEK**Sar**NQSLAFIRKSDE | 2277.5 | 26.85 |
| Sar9 | ISQVNEKI**Sar**QSLAFIRKSDE | 2276.6 | 13.89 |
| Sar10 | ISQVNEKIN**Sar**SLAFIRKSDE | 2262.6 | 21.30 |
| Sar11 | ISQVNEKINQ**Sar**LAFIRKSDE | 2303.6 | 13.89 |
| Sar12 | ISQVNEKINQS**Sar**AFIRKSDE | 2277.5 | 7.41 |
| Sar13 | ISQVNEKINQSL**Sar**FIRKSDE | 2319.6 | 4.63 |
| Sar14 | ISQVNEKINQSLA**Sar**IRKSDE | 2243.5 | 4.63 |
| Sar15 | ISQVNEKINQSLAF**Sar**RKSDE | 2277.5 | 30.56 |
| Sar16 | ISQVNEKINQSLAFI**Sar**KSDE | 2234.5 | 0.00 |
| Sar17 | ISQVNEKINQSLAFIR**Sar**SDE | 2262.5 | 84.26 |
| Sar18 | ISQVNEKINQSLAFIRK**Sar**DE | 2303.6 | 21.30 |
| Sar19 | ISQVNEKINQSLAFIRKS**Sar**E | 2275.6 | 59.26 |
| Sar20 | ISQVNEKINQSLAFIRKSD**Sar** | 2261.38 | 100.00 |
