# Supplementary material for: A Readily Applicable Strategy to Convert Peptides to Peptoid-based Therapeutics
Source: PLoS One. 2013 Mar 21;8(3):e58874. doi: 10.1371/journal.pone.0058874 (PMC3605428; doi:10.1371/journal.pone.0058874)
Supplement: Methods S1 — Additional experimental methods. (DOC) [file pone.0058874.s006.doc]

**Additional experimental methods**

**Stock solution preparation.** In the case of using dimethyl sulfoxide (DMSO) for dissolving the peptides in a high concentration (e.g., > 5 mM), immediate use is recommended. At room temperature, peptide stock solutions in DMSO appeared to be clear with no sign of precipitation. However, after being stored at 4 °C, the solutions may become frozen due to low melting point of DMSO (18.5 °C) and stay turbid and viscous, even after being incubated at room temperature for over 1 hr. This will ultimately interfere with polarization measurement. To overcome this solubility issue, storage of the stock solutions at 4 (~ days) or -20 °C (~ weeks) in 50% of acetonitrile is recommended.

**Sample preparation for FP measurements.** Due to the relatively high concentrations (100 and 200 μM) used in the 5HB-based competitive FP assays, precipitations were often observed. To reduce loss of peptides due to their poor solubility, vortexing and/or sonication were carried out. In addition, to minimize undesirable perturbation in polarization measurement caused by remaining precipitates, the solution of each sample was spun down and then transferred to the 96-well plate with care, followed by 10 sec delay before measurements.

**Monomer preparation: 3-Azidopropylamine** [1]. Synthesis of 3-azidopropylamine (2) is shown in Scheme A1 below. Commercially available 3-chloropropylamine (1) is refluxed with 3 eq. of sodium azide in water at 80 °C overnight to complete the conversion of (1) to (2). The reaction was then cooled and treated with NaOH (10 M) until pH became basic. The aqueous solution was extracted using chilled ethyl ether three times and then the combined organic layer was dried over Na_2_SO_4_, and filtered. Due to the low boiling point of the desired product, the organic solution was concentrated under weak N_2_ flow for over 1 hr. Resulting 3-azidopropylamine and H^1^ NMR confirmed that (2) was obtained with reasonable purity. After concentration was determined, (2) was diluted in *N*-Methylpyrrolidone (NMP) as a final concentration of 1.0 M for peptoid synthesis. ^1^H NMR was used to confirm the synthesis and to determine the concentration of the solution; solvent was further evaporated if necessary. ^1^H NMR (500 MHz, CD_3_OD) δ 3.40 (t, *J* = 7.0, 2H, N_3_CH_2_), 2.79 (t, *J* = 7.0, 2H, H_2_NCH_2_), 1.77 (m, *J* = 7.0, 2H, CH_2_CH_2_CH_2_) (data shown in Figure A1).

**Scheme S3.** Synthesis of 3-azidopropylamine (2) from 3-choloropropylamine (1).

**Figure S3.** ^1^H NMR of 3-azidopropylamine, (500 MHz, CD_3_OD) δ 3.40 (t, *J* = 7.0, 2H, N_3_CH_2_), 2.79 (t, *J* = 7.0, 2H, H_2_NCH_2_), 1.77 (m, *J* = 7.0, 2H, CH_2_CH_2_CH_2_)


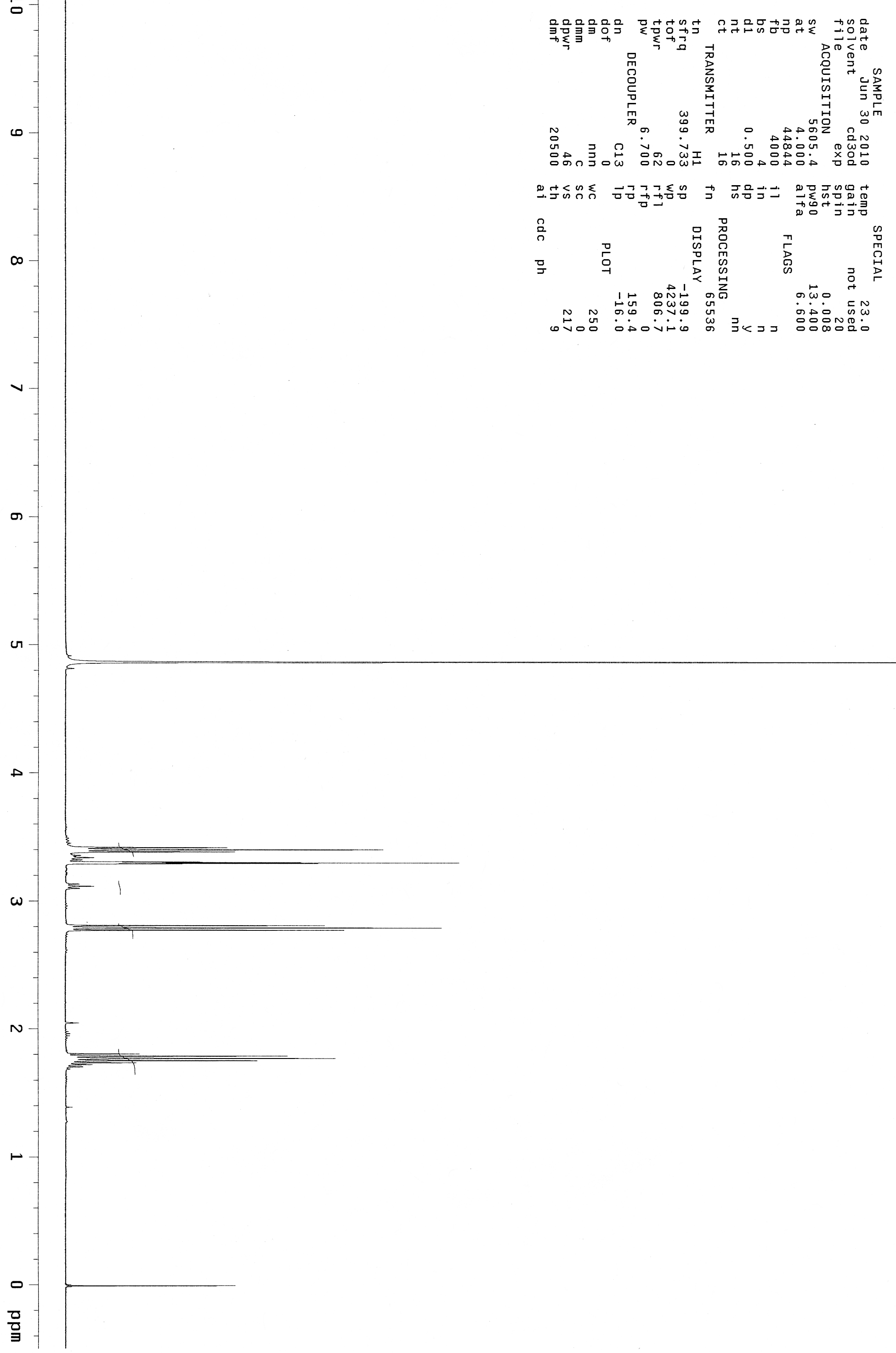


**Modified Kaiser method.** The completion of the click reaction can be monitored by various methods including the growth of a new band at  1684 cm^−1^ in IR (infrared) spectroscopy corresponding to the triazole ring [2], with HPLC analysis with retention time (Rt) difference [3, 4], or a modified Kaiser method [5]. Because the click reaction in this study was performed on resin, IR spectroscopy would not be effective. We thus decided to use a modified Kaiser method, which utilizes triphenyphospine (TPP) to reduce unreacted azide to amine and subsequently, use ninhydrin to detect free amine group [5]. 200 μL of 5% TPP was added on to the peptomer-bound resin and then the solution were vortexed, followed by the addition of 5% ninhydrin in ethanol. The mixture was heated at 130 °C till the strong colors showed up (dark purple). Unclicked peptoid-bound resin and resin only were also tested as controls. For best results, it is highly recommended that TPP solutions should be prepared prior to use and/or stored under inert atmosphere to minimize air oxidation.

**References**

1 Carboni B, Benalil A,Vaultier M (1993) Aliphatic Amino Azides as Key Building-Blocks for Efficient Polyamine Syntheses. J. Org. Chem. 58: 3736-3741

2 Huang X, Huang X-J, Yu A-G, Wang C, Dai Z-W,Xu Z-K (2010) “Click Chemistry” as a Facile Approach to the Synthesis of Polyphosphazene Glycopolymers. Macromol. Chem. Physic.

3 Holub JM, Jang H,Kirshenbaum K (2007) Fit to be tied: conformation-directed macrocyclization of peptoid foldamers. Org Lett 9: 3275-3278

4 Le Chevalier Isaad A, Papini AM, Chorev M,Rovero P (2009) Side chain-to-side chain cyclization by click reaction. J. Pept. Sci. 15: 451-454

5 Punna S,Finn MG (2004) A convenient colorimetirc test for aliphartic azides. Synlett 1: 99-100
